# Content and process: organizational conflict and decision making

**DOI:** 10.3389/fpsyg.2023.1227966

**Published:** 2023-11-14

**Authors:** Vibha Gaba, John Joseph

**Affiliations:** ^1^Entrepreneurship and Family Enterprise, INSEAD Singapore, Singapore, Singapore; ^2^Strategy and Entrepreneurship, University of California, Irvine, Irvine, CA, United States

**Keywords:** Carnegie perspective, conflict, decision making, organizational structure, slack, coalitions, sequential attention

## Abstract

The foundational work in the Carnegie perspective established conflict as endemic to organizations and a driver of organizing behavior and decision making. Organizations as a system of coordinated action among interdependent individuals and groups with different preferences, interests, information, or knowledge create the potential for pervasive and ongoing latent goal conflict. At the same time, extant psychology research has devoted considerable attention to identifying the content and intensity of conflict, focusing on the relationship between different types of conflict and their impact on group outcomes. The Carnegie perspective also assumes that the need for joint decision-making and the differences in goals or perception of reality are never fully resolved. As a result, it has paid attention to the processes through which conflict is addressed - by attending sequentially to goals, decentralizing information, accumulating excess resources, and forming coalitions rather than formal mediating procedures. The assessment of the psychology and organizational theory research also suggests that future work focusing on organizational conflict as latent, situated, and dynamic would enable greater clarity on how organizations make decisions.

## Introduction

The Carnegie perspective established intra-organizational conflict as a fundamental issue for organizations and a driver of organizing behavior, information processing, and decision making (Joseph and Gaba, 2020). March and Simon (1958) defined conflict as the “*breakdown in the standard mechanisms of decision-making so that an individual or group experiences difficulty in selecting an action alternative”* (p. 132). An important insight from this research is that when multiple preferences and goals must be addressed simultaneously, the potential for organizational conflict arises. Conflict can interact with various other behavioral mechanisms, such as aspiration adaptation, problemistic search, and attention allocation, making it a central concept for a behavioral theory of decision making, and the Carnegie perspective more broadly.^1^

Conflict is also a central concern of psychology, but this research has differed from that in organizational theory and strategy. Although early psychology work on conflict shared the BTOF’s focus on goal conflict, the emphasis changed in the 1990s. The psychology literature largely relegated goal conflict to the background and focused on situations where goals were shared, but conflict still existed (Jehn, 1995). The subsequent stream of psychology research on conflict – which follows from perceived incompatibilities or differences among group members (De Dreu and Gelfand, 2008) – focuses on identifying the content and intensity of conflict. This work made construct validity and empirical testing a priority. It focused on the relationship between three types of individual-level conflict – task, relationship, and procedural conflict—their expression (Weingart et al., 2015) and their impact on group outcomes (e.g., Jehn and Mannix, 2001; De Dreu, 2006; Jehn et al., 2008). This research is concerned with accurately measuring different types of conflict and the contingencies that promote or limit conflict’s use as a problem solving and information processing mechanism (cf. de Wit et al., 2012).

However, far from being an organizational problem that needs “solving,” conflict in the Carnegie perspective is viewed as an inherent characteristic of organizations that is never fully resolved (Cyert and March, 1963, p. 75). This perspective emphasizes that the generation and presence of conflict are endemic to organizations and that conflict is inevitable in multiple-actor decision-making processes. As Pondy relates, “Organization is a means for internalizing conflicts, for bringing them within a bounded structure so that they can be confronted and acted out. Suppose we treat organizations as arenas for staging conflicts and managers as fight promoters who organize bouts and as referees who regulate them. Far from being a “breakdown” in the system, conflict … is the very essence of what an organization is.” (Pondy, 1992).

As a result, the focus of research based on the Carnegie perspective has been the conflict-related processes that link multiple goals, aspiration levels, and performance with behavioral and decision-making outcomes (cf. Shinkle, 2012). For example, in the many studies that connect performance feedback with decision making, the generative mechanisms are described as expressions or functions of latent conflict. Also, the specific responses to feedback are primarily attributed to some form of conflict resolution (e.g., goal prioritization, interactions between subunits, or utilization of slack). Throughout this stream, the content of the conflict has remained constant (that of goal conflict). However, the emphasis on process – a hallmark of the Carnegie perspective – and the study of the empirical regularities in decision-making - not only focuses on a particular decision in time but also connects decisions over time as situational or contextual factors evolve and adapt (Beckman, 2015).

In what follows, we attempt to highlight the process-oriented lens of the Carnegie perspective, which may offer psychology scholars new directions for their theories of conflict. To do so, we first examine the role of conflict in the foundational works of the Behavioral Theory of the Firm. We pay particular attention to the processes that generate ongoing latent conflict. We consider the conditions where conflict occurs and the implications for organizational decision-making. Second, we examine the processes by which the Carnegie perspective allows organizations to function even amidst perpetual latent conflict. These processes are not meant to eliminate conflict but to promote the efficient and effective functioning of the organizations, even as tensions continue to exist. Third, we feature three characteristics of conflict that both organization theory and psychology scholars have more recently recognized as important for advancing insights into conflict as an organizational phenomenon and as a determinant of decision making: conflict as latent, contextual, and dynamic.

## Conflict in the behavioral theory of the firm

A central contribution of the Carnegie perspective is the view of organizations as shifting political coalitions with different goals and characterized by latent organizational conflict. This perspective presents a theory of organizational decision making that highlights the organization as a system of coordinated action among interdependent individuals and groups with different preferences, interests, information, or knowledge. The view is directly concerned with the contributions made by members of the political coalition to the firm’s survival and the observation that the political coalition must continually negotiate coalition membership and the subgoals that define the priorities of the firm. As such, they recognized that the stability of the coalition is fragile and that latent conflict is always present.

Although central to Carnegie perspective, the subject of conflict receives unequal treatment in foundational work. Each contribution focuses on different aspects of conflict and different features of conflict resolution. For example, Simon (1947) emphasizes conflict based on different goals and imperfect knowledge. He says, “Discrepancies arise out of the cognitive inability of decision making to deal with the entire problem as a set of simultaneous relations” (p. 160). Resolution is based on the division of labor (specialization) and corresponding local rationality associated with subgoal attention, leaving the conflict to be resolved through the hierarchy of authority. Hierarchical authority gives implicit weight to various demands in decision making and prioritizes overall organizational well-being.

March and Simon (1958) emphasize organizations as settings where individuals and groups with different goals participate in organizational decision making. The potential for conflict within an organization is expected to vary with not only differences in goals but also task interdependencies among coalition members and variations in the perceptions of the organization and its environment. Problem solving, persuasion, bargaining, and politics are likely to emerge as solutions to organizational conflict. The solution depends on whether goals are assumed to differ, and the extent to which agreements reached must be public. However, in any case, it must involve some form of continuous negotiation. Relatedly, the formation of coalitions – specifically, which coalitions are likely to form and how stable these coalitions are likely to be – is reflective of how decision-makers respond to conflict.

Cyert and March (1963) further develop the idea that organizations are shifting political coalitions and focus on the processes by which individuals with different goals participate in organizational decision making. Conflict is persistent because of the inability to write complete contracts (Pitelis, 2007) and because diverse interests and attention patterns do not allow for the full adjudication of different policy demands on the organization and require the continuous negotiation of such demands. Further, they offer a quasi-resolution of conflict through the decentralization of decision making and goal attention, sequential attention to goals, and adjustment in organizational slack. Through these mechanisms, the organization yields decisions that accommodate potential conflict.

Notably, while Cyert and March’s (1963) *Behavior Theory of the Firm* formalizes the generative processes behind joint decision making and conflict at the *organizational level* only, March and Simon’s (1958) *Organization* elaborates on both individual and organizational decision making. The literature has primarily proceeded along separate lines, with some studies focusing on individuals (e.g., Mount and Baer, 2022) and others theorizing group or aggregated decision-making processes (e.g., Bromiley, 2009).

## Antecedents of latent conflict

Of particular interest to Carnegie scholars are the conditions needed for the presence of conflict: *the need for joint decision making* and *differences in goals* or *differing perceptions of reality* (March and Simon, 1958, p. 156). These differences are not fully resolved, so pervasive latent conflict persists in organizations (Cyert and March, 1963, pp. 214–215). According to the Carnegie perspective, joint decision-making is needed when individuals, groups, or subunits face interdependent activities and must coordinate to achieve a unity of effort (Lawrence and Lorsch, 1967; Thompson, 1967). Joint decision-making is also often called for when subunits share a common pool of resources and are linked in the resource allocation process (e.g., Joseph and Wilson, 2018). Studies using a Carnegie perspective lens have operationalized this joint decision-making in terms of interdependent goals or aspirations (e.g., Hu et al., 2017), performance feedback (Joseph and Gaba, 2015) as well as the rules governing those interactions (Siggelkow, 2002; Rivkin and Siggelkow, 2007; Albert et al., 2015).

### Differences in goals

Difficulties in joint decision-making arise in the presence of multiple goals. According to the Carnegie perspective, organizational decision-makers face an array of goals when making choices. Multiple preferences within the firm form organizational goals and determine how the attention and energy of decision-makers will be allocated based on those preferences. For example, conflict may arise between the corporate headquarters and constituent subunits, with the corporate office focusing on goals related to the performance of the entire enterprise and subunits with their parochial interests and goals, which can lead to tension between the two (Gaba and Joseph, 2013). Also, conflict may arise between divisions of a large multidivisional firm (Vissa et al., 2010; Arrfelt et al., 2013). As Hu et al. (2017) remark, “Divisions and division managers in multidivisional firms tend to differ in preferences and goals, resulting in internal conflicts within the firm” (p. 1438). Organizational subunits with distinct functions are expected to develop their own goals and compete for scarce resources with other units, even though they must cooperate to support decisions. Subunits may engage in social comparisons that play up their strengths (and discount their weaknesses) vis-à-vis other subunits (Jordan and Audia, 2012; Kacperczyk et al., 2015; Baumann et al., 2019), which can serve as a source of unchecked tensions.

Compounding these issues is the observation that goals are often correlated (Ethiraj and Levinthal, 2009; Gaba and Greve, 2019). Although correlated or interdependent goals may be congruent and mutually reinforcing (positively correlated), they are more commonly divergent, whereby one goal’s satisfaction comes at the expense of achieving one or more other goals (negatively correlated). By extension, decision-makers may face negatively correlated aspiration levels and feedback (Joseph and Gaba, 2015). Problem-solving behavior is, therefore, affected by the goals on which decision-makers choose to focus and the interdependencies among those goals.

The complexity associated with multiple interdependent goals may create difficulties in establishing decision-making criteria (Sundaram and Inkpen, 2004). Multiple goals may create tensions over the direction of the organization and, therefore, fuel ongoing debate and produce delays. Thus, coordination challenges are significant in the presence of multiple goals (Hu and Bettis, 2018; Audia and Greve, 2021). Problems associated with multiple goals are likely to increase with the number of goals a firm pursues (Ethiraj and Levinthal, 2009), as each additional goal substitutes effort and attention from previously established goals (Stevens et al., 2015). This was confirmed empirically by Obloj and Sengul (2020), who found that the likelihood of increased performance on any given performance dimension decreased with the number of other concurrently pursued goals. However, they also found that this applies to most but not all goals. The multiplicity of objectives negatively impacts market share, cost, and export goals but not revenue and margin goals, which are presumed to have a comparatively lower level of interdependence with other goals and ostensibly limit ongoing tensions.

Various studies have examined the processes associated with the direct or indirect effects of goal interdependencies. For example, Gaba and Greve (2019) consider the airline industry’s dual focus on safety and profitability and how it affects decisions regarding fleet changes. In the airline industry, safety and profitability have clear conflicts (at least in the short term) owing to the costs of replacing aircraft models with poor safety records. They show that the pursuit of safety goals cannot be understood in isolation from profitability goals, and responsiveness to safety goals is strengthened by low profitability. The reason is that performance shortfalls on multiple goals can trigger survival concerns, leading decision-makers to respond to goals differently. In such situations, the goal perceived as essential for survival gets priority and triggers stronger reactions. In their study, responsiveness to safety goals is strengthened by low profitability because low safety means a risk of accidents, which could lead to organizational failure. Their work suggests that managerial focus on survival rather than shifting attention among multiple goals is another approach to reconciling goal conflict.

Hu and Bettis (2018) study three product-level goals (safety, efficiency, and reliability) with shared technological task environments in the automobile industry. In their study, goal fulfillment becomes interdependent because of a shared task environment. As a result, actions in one task environment to improve the performance of a particular operational goal can simultaneously impede or enhance the performance of other operational goals in the same task environment. They conclude that such interdependencies can lead to severe confusion and stall the coordination efforts, further complicating the problem-solving process. Although not a primary focus, they recognize that in such environments, assigning credit will be increasingly cognitively intractable (Minsky, 1961), leading to potential conflict and disruption of response to feedback.

Salvato and Rerup (2018) examine the conflict arising from the simultaneous pursuit of design and efficiency goals in new product development. Here, they highlight March and Simon’s (1958) emphasis on performance programs (or routines) theorized as a stable pattern of action, predictably performed and oriented toward a particular organizational goal. However, they also highlight that the conflict-mitigating benefits of these programs or routines break down in the presence of multiple goals. That is, while the individuals enacting a program to support a particular goal implicitly consent to perform their role and enact a truce (Nelson and Winter, 1982), multiple goals render the truce through routine ineffective.

Organizations are embedded in various industry and institutional contexts, and each may impose goals that are likely to be cognitively available in the course of decision making along with the internal goals (Gavetti et al., 2007; Greve and Teh, 2018; Keum and Eggers 2018; Joseph and Gaba, 2020). For example, Rowley et al. (2016) examined the conflict arising from externally imposed goals, such as public ratings and rankings, the pursuit of which can potentially divert resources from internally established goals. Because rating systems can positively or negatively affect firms’ reputations, it can increase organizational pressures to adopt changes that such ratings are designed to promote. They study the adoption of governance practices in response to performance feedback on both financial profitability and position on a governance rating and find that firms with both poor profitability and poor performance on the external governance rating are least likely to adopt the rating-consistent practices. Their study suggests a hierarchical ordering of goals to resolve conflict, reflecting decision makers’ choices on which problems to pursue.

In related work, Birkinshaw and Lingblad (2005) find that the potential for intra-organizational competition increases with the extent to which subunits have overlapping intra-firm charters. Charters are the technologies, products, or customer groups the subunit is oriented toward and the organizational domain the subunit has staked out. While such overlap can serve as a source of motivation, it can also serve as a source of conflict as coordination costs and the battle for resources increase. Similarly, Joseph and Wilson (2018) found that intra-organizational conflict can occur between separate subunits (that are horizontally coupled) who compete for similar internal (e.g., corporate) and external (e.g., customer) resources with different technologies; the sales of one unit’s output may be threatened by the output from other units. Making the distinction between *coordinative tensions* – intra-firm conflicts over routines and activities – and *cooperative tensions* – intra-firm conflicts over resources and control, they note that when the coupling is tight and the actions are directed toward similar objectives, the conflict that arises stems from differences in opinion about resource allocation and decision-making control (i.e., autonomy).

Another source of conflict may stem from the variation in aspiration levels that firms compare themselves to and the performance feedback they receive. Joseph and Gaba (2015) recognize that feedback from these comparisons may be consistent, inconsistent, or ambiguous. Divergent aspiration level comparisons can reflect different social and historical aspirations (Baum et al., 2005; Lucas et al., 2018), forward- and backward-looking aspirations (Chen, 2008), internal and external social aspirations (e.g., Kacperczyk et al., 2015; Hu et al., 2017; Baumann et al., 2019), short-term and long-term aspirations (e.g., Cheng et al., 2022), and aspiration comparisons over time (Joseph and Gaba, 2015). Divergence may occur even amidst consistent performance feedback – for example, *peer* overperformance can demotivate search, but *historical* overperformance can motivate search (Ye et al., 2021).

Both inconsistent and ambiguous feedback can distort performance assessment and decision-making processes, which, in turn, may increase internal tensions. First, dual comparisons can confound information processing and shape decision-making in terms of search and performance (Baumann et al., 2019), as well as change and risk-taking (Kacperczyk et al., 2015). For example, conflict can arise when managers face divergent feedback from comparing performance to two different social aspiration levels – that of external peers and that of other internal divisions or what is known as a political reference point (Hu et al., 2017).

Second, decision-makers may disagree on whether there is a problem to begin with. Accordingly, conflict arises because poor performance prompts problem-solving efforts in some but self-enhancing behavior in others (Audia and Brion, 2007; Jordan and Audia, 2012; Audia et al., 2015). Self-enhancement refers to the interpretation of performance in a favorable way and has been shown to hinder problemistic search (Argote and Greve, 2007; Kacperczyk et al., 2015; Lv et al., 2019). At the same time, managers will take advantage of feedback inconsistency and attribute problems to factors beyond managerial control, negatively impacting problemistic search (Arrfelt et al., 2013), putting those decision-makers at odds with those attempting to find real solutions to problems. This dynamic is contingent on factors such as different types of CEO power (Blagoeva et al., 2020), family control (Lv et al., 2019), and stakeholder demands (Dye et al., 2014).

Third, if decision-makers agree that a problem is present, they may disagree on the appropriate response, increasing divisiveness. Inconsistency in performance feedback can lead to an intense and ongoing debate among decision-makers concerning the appropriate solution (Joseph and Gaba, 2015; Desai, 2016) and interfere with alternative selection and implementation (Cyert and March, 1963).

### Differences in perceptions

Conflict may also arise when differences in perceptions of the internal or external environment occur or when perceptions of performance feedback and objective performance measures differ (Saraf et al., 2022). Researchers interested in cognition (cf. Posen et al., 2018) note that organizational members develop cognitive representations of an organization’s internal and external environment, referred to as representational complexity (Csaszar and Ostler, 2020) and assumed interaction structure, variously interdependence representations (Martignoni et al., 2016) or logics of organizing (Alexy et al., 2021). However, these representations may or may not align – with differences persisting between individuals and across the organization. Differences have implications for variation in approaches to problemistic search and related decision-making.

Managers will find it easier to cooperate if their perceptions reflect the similar encoding of the internal and external environment. Shared conceptions of problem-solving activities have been argued to be an essential mechanism for coordination within the firm (Okhuysen and Bechky, 2009; Leonardi, 2011) and may keep conflict within an optimal range (Eisenhardt and Schoonhoven, 1990; Jehn and Mannix, 2001). This accords with research arguing for overarching strategic goals or direction (Stieglitz and Heine, 2007; Leiponen and Helfat, 2010; Gulati et al., 2012a) and models of shared cognition that focus on the performance implications of broadly diffused mental models, schemas, frames, and logics (Eggers and Kaplan, 2009, 2013).

However, differences may exist between perceptions of performance feedback and objective measures of performance (Saraf et al., 2022). Individual cognitive representations often differ from the true underlying interdependence structure (e.g., interactions among internal activities or goals) or external complexity (e.g., interactions between the firm and its environment), as well as differ between individuals or groups. Differences in individual or shared group perceptions stem from a variety of sources: political processes (Tarakci et al., 2014; Hu et al., 2017), changes in the competitive environment (Porac and Thomas, 1990), regulatory focus (Higgins, 1998; Gamache et al., 2015), cognitive frames (Osiyevskyy and Dewald, 2015) and location in the organization (Gavetti, 2005; Vissa et al., 2010; Gaba and Joseph, 2013; Rhee et al., 2019).

Differences in perceptions based on location may differ because (1) Problem-solving is motivated in areas considered important by decision-makers and their constituents, which may differ with the individual’s role in the organization (e.g., senior vs. lower-level managers, engineering vs. marketing, line vs. staff); (2) Problem-solving is shaped by managers’ experiences and by the information available to them for decision making. Different roles bring different biases to problem-solving efforts, shaped by each manager’s background and experience (Gaba et al., 2023). (3) Problem solutions are sought “near the symptoms,” meaning that individuals with different causal models may disagree on the cause of and solutions to the problems (Gaba and Joseph, 2013). For example, lower-level roles reflect simple or parochial causal models, motivating local search. More senior roles may reflect more complex cognitive models, which reflect interdependent problems that senior managers must manage simultaneously. As a result, these managers seek broader solutions that cover various problems across the organization.

Illustratively, Joseph and Wilson (2018) document the conflict that arose at Motorola due to different perceptions of new technology. The corporate office and the division dedicated to the new technology saw the technology as an opportunity. However, one of the legacy units viewed the technology as a threat, which fueled conflict over whether and how the new technology should be developed. Research shows that more favorable perceptions will weaken the impact of negative performance feedback on problemistic search and, consequently, decision making (Saraf et al., 2022).

## Processes of conflict resolution

The BTOF and its progeny (e.g., organizational learning, attention, performance feedback) have dedicated much scholarly effort to the processes for resolving conflict. Herein lies the significant potential for the Carnegie perspective to add unique value to other psychological theories of conflict. While psychological studies mainly establish or manipulate goal alignment to focus on the implications of different types of conflict, the Carnegie perspective assumes that true goal alignment never occurs. The theory assumes that “except at the operational level, there is no internal consensus. The procedures for “resolving” such conflict do not reduce all goals to a common dimension or even make them internally consistent” (Cyert and March, 1963, p. 117). Given the pervasiveness of intra-organizational conflict, the focus is not on explicit mediation procedures to resolve conflict. Instead, organizations tend towards a quasi-resolution of conflict, “the tendency of organizations to address different goals through coalitions that represent temporary compromises between different goals” (Gavetti et al., 2012, p. 6). Conflict is ameliorated by attending sequentially to goals, decentralizing information, accumulating excess resources, and forming coalitions (March and Simon, 1958; Cyert and March, 1992). In that sense, the theory aims to provide a process-oriented and more behaviorally plausible account of organizational decision-making.

### Sequential attention

Foundational BTOF work establishes that to deal with the cognitive burden and potential discord commonly associated with multiple goals (Jensen, 2002; Sundaram and Inkpen, 2004; Ethiraj and Levinthal, 2009), attention to goals will be sequential (Greve, 2008; Gaba and Greve, 2019). Sequential attention is the idea that to process multiple goals, decision-makers switch their attention back and forth between them (March and Shapira, 1992; Stevens et al., 2015). Many of these goals are assumed to be essential, continuous, and operative, which means they can pose problems—in the form of potential conflict—for the organization.

Goals are evoked and pursued when performance problems or attainment discrepancies arise; consequently, they motivate action toward resolving the most pressing problem or gap between performance and a particular goal (Greve, 2003). When performance on a particular goal is above the aspiration level, decision-makers move on to the next goal, which requires attention and action (Cyert and March, 1963, p. 117–119). Behavioral researchers have tested this sequential attention assumption and found that low performance on a lower-priority goal spurs reactions only when performance on a higher-priority goal signals success (e.g., Greve, 2008; Stevens et al., 2015). As Greve (2008, p. 480) noted, “Sequential attention is a form of quasi-resolution of conflict that lets decision-makers treat different goals as constraints to be satisfied in some order of priority rather than as tradeoffs that need to be weighed against each other. It reduces cognitive effort and political strife and thus yields easier, but possibly suboptimal, organizational decision making.”

The capacity for sequential attention to alleviate potential tensions comes in many forms. When contrasting signals are present – decision-makers may often lean more heavily on those that provide clearer signals/information. Managers focus on dimensions with more concrete causal implications. For example, Zhang and Gong (2018) find that prior years’ sales growth provides clearer signals than prior years’ stock market returns to managers regarding the firm’s standing in the product market and customer and competitive information. Another pattern of sequential attention is that firms focus on the short term over the long term. Feedback from short-term goals is likely to provide clearer signals about a logical course of action when performance is below aspirations and external oversight is high (Cheng et al., 2022).

Sequential attention may also come in the form of focusing on historical or social aspirations rather than both at once. Research shows that attention to social and historical aspirations can vary over time, with more attention going to historical aspirations in turbulent environments due to the lower information requirement (Greve, 2003) and more attention to social than historical when decision-making is higher in the organization – or more centralized – due to greater demands from and attention to the external environment (Joseph and Wilson, 2018; Dutt and Joseph, 2019; Berchicci and Tarakci, 2022). Sequential attention may also be resolved by creating routines that offer opportunities to address multiple goals at once. Salvato and Rerup (2018) found that by creating routines that offered opportunities to focus on a particular goal while lowering the contention among decision-makers, the organization could reduce conflict and allow the new product development process to proceed.

These studies on multiple goals and aspirations are important because they highlight how organizations problem-solve through goal prioritization, managerial focus, and the temporal separation of goals. However, this work is also based on the strong assumption that goal prioritization is stable and uncontested (Greve and Gaba, 2017). It also does not account for what happens when multiple goals are difficult to prioritize or signal conflicting courses of action, whereby the satisfaction of one goal comes at the expense of achieving one or more other goals. Likewise, the role of the external environment in providing goals and when these goals conflict with internally established goals needs more attention. Given the ubiquity of goal conflict in organizations, much more work is needed to understand the subtle connections between goal conflict and complexity and the tradeoffs by which such conflicts are resolved in organizations.

### Specializing and differentiating through structure

Another way to deal with conflict that may arise from multiple goals is through the organizational structure. Organizational structure –the division of labor and specialization – focuses decision-maker attention on problems and subgoals corresponding to organizational subunits, divisions, departments, or groups (March and Simon, 1958; Cohen, 1984; Ethiraj and Levinthal, 2009).

The division of labor is effective at conflict mitigation because it establishes a local rationality of decision-making in that individuals will deal with only a limited set of problems (subgoals) at a time (at the limit, one each). The advantage stems from simplifying the decision-making environment for subunit managers and limiting the cognitive complexity associated with many interacting goals and activities. Narrowing cognition will lead to a corresponding change in the nature and interdependencies of subgoals, reflecting the dynamics of organizational structure. The division of labor can occur along the vertical hierarchy between the corporate office and constituent subunits (e.g., Gaba and Joseph, 2013) or across multiple subunits or groups (Vissa et al., 2010; Rhee et al., 2019). Accordingly, attention allocation, aspiration formation, search processes, and responses to performance feedback will also vary.

Such divisionalization benefits the organization by confining most interdependencies within self-contained subunits and minimizing the interdependencies between them. As a result, local decisions need to satisfy local demands only. In this way, differentiation keeps the perturbations in one unit from negatively affecting other units (Fang and Kim, 2018). By decomposing the organization and corresponding subgoals, unit managers may readily acquire, process, and utilize information necessary for achieving those subgoals while limiting the impact of any disruptions to the unit on other units.

For example, Joseph and Wilson (2018) found that explicit conflict between division heads and the demands to resolve the tension caused the separation of organizational units. In particular, two interdependent subunits were competing for similar internal (e.g., corporate) and external (e.g., customer) resources with different technologies, and the achievement of one unit’s goal was threatened by the other unit, which led to the separation of the units. The overall effect of separating the subunits was to transform destructive *intra-organizational* conflict into constructive *inter-organizational* competition. For example, the separation allowed each subunit to focus on its respective technologies even though their market opportunities were similar. The separation also alleviated the attention load of the legacy unit and allowed their managers to focus on previously underemphasized aspects of their technology and customer base.

Another structural mechanism to deal with conflict resolution is the organizational hierarchy. Firms may vest decision-making authority with subunits at higher levels of the corporate hierarchy to alleviate conflict, which has several implications (benefits) for managing multiple goals. First, the corporate hierarchy is less concerned than divisional or functional managers with any single performance dimension. For corporate executives, the performance of any one business, product, or technology is less critical to assessments of their performance, so when the subunit experiences a failure in one domain, belief in the attractiveness of alternatives may be relatively more favorable. As a result, they will be more willing in general to abandon failures (Eggers and Kaul, 2018; Joseph et al., 2018) and redirect resources toward successes. Also, corporate managers have the full remit to reallocate resources. They have the authority and capacity to redirect resources from unsuccessful to successful markets and avoid internecine feuds.

Second, vesting decisions at higher levels allows for a shift of attention to a broader range of goals. Such a shift of attention reflects the need of firms to meet the aspirations of different supporters (Ahn et al., 2017; Kotlar and Chrisman, 2019). As the composition of interest groups becomes diverse, firms attempt to adjust the aspiration level regarding a broader range of goals to avoid conflicts between groups (Greve, 2008; Vissa et al., 2010). Although such a shift inhibits the firm’s response to serious negative feedback and weakens the role of negative feedback in triggering problemistic searches – it may avoid the deleterious effects of conflict.

Third, corporate executives or other high-status individuals are likely to be more responsive amidst conflicting goals (Kostopoulos et al., 2023). High-status decision-makers can better search within a broader solution space and initiate more changes when experiencing poor performance because of their access to resources and opportunities. Moreover, the status may work against their propensity to self-enhance amidst conflicting goals and reduce perceptions of threat when performance is below aspirations on a primary goal (and above on a secondary goal) that gives rise to self-enhancement. Further, diversification provides executives a means to self-enhance – to focus on corporate performance if subunit performance is poor or vice versa (Lim and Audia, 2020).

### Utilization of slack

Slack is the third mechanism used to alleviate intra-organizational conflict. Slack is defined as the “use of administrative resources beyond what is necessary for the short-term operation and maintenance of the organization” (Greve, 2003, p. 688) and typically follows from higher performance (Titus et al., 2022). The Carnegie perspective articulates multiple benefits of slack (Bourgeois, 1981). Although agency theorists and organizational economists associate slack with inefficiency (Williamson, 1963, 1964; Jensen and Meckling, 1976), slack is a mechanism by which the political coalition of the firm maintains power. Because slack reflects the difference between resources needed to maintain routine operations and the resources received by a coalition in the organization, slack can be used to mitigate conflict that naturally arises from scarcity. Projects with different goals can coexist because resource competition is less intense (Kuusela et al., 2017). Moreover, a single project can accommodate more demands, alleviating concerns that “pet projects” will not be funded. Slack lowers the chance that an organization would have to take unwanted actions (George, 2005), reducing opportunities for tensions to develop.

By extension, slack – because it is typically present in successful organizations—can help fuel search and innovation. For example, research shows that slack increases R&D intensity because it allows for pursuing projects, although this research usually omits direct references to conflict (Greve, 2003). Slack moderates firm responses to performance shortfalls and can lead to higher investment in innovative outcomes following performance below aspirations.

Slack can lead to more novel and risky actions (e.g., Greve, 2003; Baum et al., 2005; Baum and Dahlin, 2007; Chen and Miller, 2007) and exploration, especially when there is an environmental threat (Voss et al., 2008). By buffering organizations from the threat of failure, slack resources permit managers to respond to low performance by increasing investments in innovative competencies (Lungeanu et al., 2016). Although, others have shown a curvilinear relationship between slack and innovation (Nohria and Gulati, 1996), R&D investments (Kim et al., 2008), and performance (George, 2005).

The creation of slack and its uses is primarily positioned as a natural outcome of bargaining and decision-making processes, and its generation and application are therefore not theorized as explicit. Cyert and March (1963, p. 44) explicitly remark that it does not arise from “conscious intent” and is used to absorb excess resources and serve as emergency resources. Moreover, its usefulness and qualities as side payments are generally expressed in terms of its impact on aspiration levels; slack has not received much consideration regarding its impact on the content of the aspirations itself. Collectively, the BTOF is unclear on when slack may be used to reduce intra-firm conflict and when it might be channeled toward other outcomes, such as innovation. That is, the foundational theory and subsequent work do not provide a clear understanding of when slack is likely to be applied toward conflict reduction by the political coalition and when it is expected to be channeled toward innovation activities. We do not fully comprehend the motivational components behind the use of slack, the conditions that enable the political coalition to maintain peace or pursue innovation, and those that will induce one action or the other. For example, such situations could be related to the type or intensity of conflict (e.g., task, process, or relationship) or whether innovation opportunities are short-term or long-term, but more work is needed.

### Coalition formation

The concept of organizational coalitions is foundational in BTOF and essential for understanding the perpetual nature of conflict and compromises in organizations. According to Simon (1964), the goal of an action is not necessarily unitary but may emerge from a series of demands the actions must satisfy. Organizational goals are thus formed by multiple demands and preferences within the firm and determine how the attention and energy of decision-makers will be allocated based on those preferences. An important implication of this view is that organizational goals are contested overall and in specific decisions, so political coalition building is essential for resolving goal conflict (Cyert and March, 1992).

Coalitions are often issue-based (March, 1994) and act as a means of political influence within the organization. Each issue has a distinct set of alternatives, and decision-makers seek to build and retain coalitions to influence decisions related to the issue. The individual participants in a coalition may have different preferences concerning those issues that may never be fully reconciled but only subjugated in anticipation of actual or potential gains (Cyert and March, 1992, p. 31). This enables reconciling incompatible preference ordering without eliminating underlying differences.

In these political models, decision-making results from exercising power and influence among the coalition participants. Coalitions divide complex and interrelated problems into a “number of simple problems,” reducing the cognitive effort of comprehending and responding to issues and controlling latent conflict between coalitions by reducing their interdependence (Cyert and March, 1963). This line of research establishes the importance of top executives as both political brokers and integrators, with neither CEOs nor other executives asserting full control over decision-making and outcomes (Zald, 1970; Pfeffer and Salancik, 1978). As issues shift, so does the power balance, such that some coalition members become more critical than others (Zald, 1962). Power accrues to those who control access to resources valued by others (Pfeffer and Salancik, 1978) and those who can resolve important contingencies facing the firm (Hickson et al., 1971).

Although empirical research on this topic remains sparse, some studies show that distinct coalitions of decision-makers may jointly influence organizational decision-making, or a dominant coalition may emerge situationally and guide responses aligned with their preferences. For example, Desai (2016) examined the joint influence of distinct coalitions of decision-makers, such as board members and managers, on organizations’ responses to the performance below aspirations. He argued that although different coalitions may vary in their preferences regarding organizational responses to poor performance, such situations increase the board’s involvement and influence in decision-making aligned with their preferences. While a dominant coalition of top managers could implement actions aligned with their priorities during routine periods, performance shortfalls force managers to seek compromises, ultimately affecting the extent of organizational change. Similarly, Greve and Zhang (2017) examined how the elements of the external environment – multiple institutional logics that embody value judgments – affect the choice of goals and connect a coalition of decision-makers with sources of support that increase its power, thereby affecting organizations’ decisions. They found that the coexistence of competing logics—state socialism and market capitalism—during China’s economic transition affected firms’ M&A decisions via the coalition building by advocates of each logic. In another study, Zhang and Greve (2019) showed that organizational coalitions could coalesce around experience-based preferences and include neutral or ambivalent members who may be recruited as allies. They further found that such coalitions strongly affected decision-making, and their solutions to organizational problems were consistent with the experienced-based preferences of the decision-making group. While these studies provide important insights into how coalitions are formed and influence organizational choices, more work is needed to understand the motivations and intentions of the coalition participants and how they structure their activities.

One omission in the Carnegie perspective is that the mechanisms to reduce conflict are assumed to have similar implications for problems of cooperation and coordination. Cooperation refers to an alignment of interests; coordination refers to an alignment of actions or tasks (Gulati et al., 2012b; Castañer and Oliveira, 2020). However, the mechanisms for conflict reduction may have different implications for cooperation versus coordination-based conflict. For example, both sequential attention to goals and structural differentiation reduce potential conflict stemming from cooperation requirements since lack of consistency between goals is not observed, and tradeoffs are not required by organizational members (i.e., they go left, and then they go right). These two mechanisms also reduce coordination-based conflict since corresponding attentional patterns mean task complexity is not observed. However, this is only true if the underlying task structure is modular (Ethiraj and Levinthal, 2004). Coordination-based conflict may be especially sensitive to reciprocal, parallel, or otherwise interdependent tasks which cannot be decomposed.

Similarly, slack has an ameliorating impact on cooperation- and coordination-based conflict but for different reasons. For the former, slack reduces potential conflict since individual demands can be met. For the latter, slack reduces potential conflict since buffers limit interdependencies. Along the same lines, coalitional behavior reduces cooperation-based conflict since the coalition may focus on goals that are non-operational (i.e., do not conflict with specific objectives) or which are held in common. Coalitional behavior may also be beneficial for coordination problems. However, in this case, it is because the coalition, operating at the highest levels of the organization, can make decisions that are mutually reinforcing and avoid conflicting courses of action. Of course, these relationships are more complex than we have articulated above and bear greater scrutiny in future research.

### Conflict as latent, contextual, and dynamic

The endemic nature of the Carnegie perspective’s treatment of conflict has been met by psychology’s greater attention to both content and process. In particular, psychology’s focus on interactions and episodes of conflict (Weingart et al., 2015; Cronin and Bezrukova, 2019) and Carnegie’s embrace of latency, situatedness, and dynamic processes point to new avenues of research for both fields and significant opportunities for cross-pollination of ideas and theory.

First, conflict in the Carnegie perspective is latent. That conflict is latent reflects initial antecedent conditions (i.e., multiple goals, scarce resources, policy debates, task interdependencies). For example, organizational theorists recognize that overt conflict is uncommon in top management teams. Despite the diverse goals, interests, and preferences among coalition members, conflict among those members is often covert, as participants selectively attend to the firm’s issues and opportunities and intermittently mobilize their power and influence (Cyert and March, 1963; Morrill et al., 2003). While explicit conflict is plausible in top management teams, much research has shown the influence of intra-organizational norms and rules in shaping elite truces (Useem, 1984; Hirsch, 1986; Ocasio, 1999; Westphal and Khanna, 2003) and standard operating procedures in limiting more widespread breakdown in the cooperative organizational system (Jehn and Bendersky, 2003).

Tensions rarely erupt in explicit conflict or spillover into visible battles. Nevertheless, internal tensions may be possible. Remarkably, much of the theorized impact of conflict is that of latent conflict, which makes conflict influential in decision-making but challenging to measure. Conflict is often “assumed” and diverges from the psychological literature, which has devoted significant attention to measurement validity and empirical research (cf. de Wit et al., 2012). Explicit conflict is rarely captured in behavioral models (much less the type of conflict involved). At the same time, psychology research’s focus on measuring direct conflict can also easily miss the underlying frictions and sources of tensions in joint decision-making with substantive impact on individual, group, and organizational outcomes. A fruitful way forward, for example, could be to measure the antecedent conditions that likely generate breakdowns as the decision-making progresses.

Second, conflict is situational in the Carnegie perspective. The BTOF established that conflict is situational and depends on various conditions to make it apparent. This aspect of conflict is perhaps the most developed in behavioral theory. It is partly due to the emphasis on situational factors to assess whether or not conflict is likely to be present. Organizations are subject to pressures from various external and internal stakeholders, some of which may conflict with each other. As noted above, new goals may emerge in the organization’s environment, creating conflict with the established internal goals, especially when the achievement of these externally generated goals requires the diversion of resources from the fulfillment of internal goals. Similarly, divergent goals or inconsistent feedback creates the context for disagreements on whether problems exist and solutions are needed. In these studies, conflict is never measured but inferred from the nature of the choice situation. Only in a few case studies (e.g., Joseph and Wilson, 2018; Salvato and Rerup, 2018) is conflict treated directly and empirically in any meaningful way. Many more quantitative studies are still needed in this vein.

The situational nature of conflict in the Carnegie perspective has meant that scholars have relied heavily on such conditions to infer the presence or absence of conflict. The focus is on goal conflict, for example, rather than the actual expression of conflict between individuals, groups, or subunits. It is important because work in psychology has recognized not only the particular type of conflict (task, process, and relationship) but also that the directness and oppositional intensity of conflict may play a vital role in the relationship between conflict and outcomes. Recent work on conflict expression recognizes that the situated nature of communication between two people and the specific content of the exchange can determine whether and how the conflict will be realized and responded to (Weingart et al., 2015). The theory foregrounds the notion that the characteristics of the conflict being experienced influence the approaches to conflict management and the outcomes.

A third aspect of Carnegie’s theorization of conflict – and a problem that it shares with the psychological literature - is that even if theorized as a process, it is often studied as a single-point measure in a variance study (Okhuysen and Richardson, 2007, p. 146). In other words, the treatment of conflict lacks process dynamics. As parties attempt to manage conflict, the nature of conflict itself changes regarding the issues considered (Carver and Scheier, 1990) and the emotions surrounding the conflict (Van Kleef and Cote, 2018). In fact, Pondy’s early approach was essentially a dynamic model of conflict, which established conflict as a series of episodes. The first was latent conflict, as described above. The second was “felt conflict,” where affective states (i.e., stress, hostility) and cognitive states (perceived conflict) were activated. At this point, conflict was salient to the individual, which led to explicit displays of aggression or resistance.

Understanding and studying conflict as a dynamic process would better allow Carnegie scholars to recognize and account for a more significant role of communication in conflict (Weingart et al., 2015) and the differences between conflict states and processes (Cronin and Bezrukova, 2019). For example, Weingart et al. (2015) offer a model that emphasizes verbal and nonverbal communication of opposition between people: the reflected directness and oppositional intensity might offer some insights as to when certain types of conflict may hurt decision-making. Understanding conflict interactions can provide information about inconsistencies and related emotions among people, which in turn influences their ability to perform tasks and resolve conflicts. Likewise, the endogenous nature of conflict relies on the feedback loop between the state and processes as the moves made to address the conflict alter the state of the conflict, and the new state then changes the subsequent processes, and the cycle continues (Cronin and Bezrukova, 2019). This view speaks to the importance of capturing the temporal dynamics of conflict, which are rarely represented in the current process frameworks of conflict studies.

A dynamic approach would also help unpack the link between conflict emerging from multiple organizational goals and decision making. In particular, we could better understand how goals are activated and used in decision-making. Theories of loose coupling suggest that organizational goals do not always affect decision-making and are used to justify action (Cohen et al., 1972; Weick, 1976; Kaplan, 2008) rather than explain purposeful decision-making (Eisenhardt and Zbaracki, 1992). From this perspective, goals are not necessarily stabilized or agreed upon before considering alternatives but are drawn from a pool of existing goals as the decision-making process proceeds. Such loose coupling would increase the possibilities for goal satisfaction (Simon, 1964) and limit the possibility of conflict. In any case, a more dynamic approach would provide a better understanding of how organization manage multiple goals.

## Conclusion

Conflict is central to the theory of organizational decision-making. The Carnegie School perspective acknowledges that organizations are not merely cooperative systems for inducing collective action toward a common purpose (Barnard, 1938); organizations are also systems of subunits headed by decision-makers who have conflicting goals and interests while competing for status and power (March and Simon, 1958; Cyert and March, 1992). In particular, the potential for conflict is greatest when there are interdependencies, differences in goals and interests, and divergent perceptions of the external and internal environment (Hayward and Boeker, 1998). Interdependences and variations in the information held (and hence perceptions) to a general sense of uncertainty introduce coordination and cooperation challenges and create latent or overt conflict within the organization (Pondy, 1967).

Although overt conflict is rare, internal tensions from divergent or ambiguous goals or feedback are likely to disrupt information-processing efficiency and learning, given that clashing managers will not be motivated to cooperate. These results are consistent with previous work on individual-level conflict, which indicates that high levels of conflict are disruptive and counterproductive to the performance of routine and creative tasks (De Dreu, 1997). Cyert and March (1963) established the so-called quasi-resolution of conflict as a central tenet of the BTOF. These authors argue for the existence of logical differences between the demands of different organizational actors and claim that dealing with the conflict inherent to these differences requires that organizations decentralize, attend sequentially to demands (goals), regulate slack resources, and form coalitions.

Both streams of work would benefit from greater attention to conflict as latent, situated, and dynamic. Such an approach may provide insights into areas that demand greater research focus, such as: What are alternatives to sequential attention to goals? When is conflict productive, and when is it not? Moreover, what motivates the use of slack resources for stabilization vs. change? In other words, approaching conflict as latent, situated, and dynamic would enable greater clarity on how individuals, groups, and organizations make decisions.

## Author contributions

All authors listed have made a substantial, direct, and intellectual contribution to the work and approved it for publication.

## Footnotes

^1^We mention the Behavioral Theory of the Firm to refer to the foundational texts: Simon, 1947; March and Simon, 1958; Cyert and March, 1963. The Carnegie perspective refers to a behaviorally plausible, decision-centered perspective on organizations which was inspired by these foundational texts and subsequently developed across a diverse set of literatures and studies.
